# Enhancing nutritional management in peritoneal dialysis patients through a generative pre-trained transformers-based recipe generation tool: a pilot study

**DOI:** 10.3389/fmed.2024.1469227

**Published:** 2024-10-30

**Authors:** Haijiao Jin, Lulu Huang, Jinling Ye, Jinkun Wang, Xinghui Lin, Shaun Wu, Weiguo Hu, Qisheng Lin, Xiaoyang Li

**Affiliations:** ^1^Department of Nephrology, Ren Ji Hospital, Shanghai Jiao Tong University School of Medicine, Shanghai, China; ^2^Department of Nephrology, Ningbo Hangzhou Bay Hospital, Zhejiang, China; ^3^Molecular Cell Lab for Kidney Disease, Shanghai, China; ^4^Shanghai Peritoneal Dialysis Research Center, Shanghai, China; ^5^Uremia Diagnosis and Treatment Center, Shanghai Jiao Tong University School of Medicine, Shanghai, China; ^6^WORK Medical Technology Group LTD., Hangzhou, China; ^7^Department of Medical Education, Ruijin Hospital Affiliated to Shanghai Jiao Tong University School of Medicine, Shanghai, China

**Keywords:** artificial intelligence, peritoneal dialysis, nutritional management, generative pre-trained transformers system, recipe generation

## Abstract

**Background:**

Patients undergoing peritoneal dialysis (PD) often face nutritional deficiencies due to inadequate intake, nutrient loss, insufficient dialysis, and a state of micro-inflammatory. Traditional nutritional management methods have not fully met personalized needs. Therefore, this study aimed to develop and evaluate an application for generating recipes based on Generative Pre-trained Transformers to improve the nutritional status of these patients.

**Methods:**

This self-controlled prospective study included 35 patients undergoing PD from January to February 2024. The study was divided into two phases: the initial phase involved conventional dietary education under PD management, followed by a second phase where a new GPT-based dietary guidance tool was introduced. Patients adhered to the diets recommended by the tool. Nutritional intervention effects were assessed by comparing serum prealbumin, albumin, and phosphate levels before and after the intervention.

**Results:**

After the intervention, the mean prealbumin levels significantly improved from 289.04 ± 74.60 mg/L to 326.72 ± 78.89 mg/L (*p* = 0.001). Although there was no statistical significance, the serum albumin levels in patients increased from 34.70 ± 5.94 g/L to 35.66 ± 5.14 g/L (*p* = 0.153). Serum phosphate levels remained stable and within safe limits (*p* = 0.241).

**Conclusion:**

The AI-based recipe generation application significantly improved serum prealbumin levels in PD patients without causing adverse changes in phosphate levels, confirming its efficacy and safety in nutritional management for these patients. This study highlights the potential and practical value of AI technology in nutritional management for patients with chronic disease, providing important evidence for future clinical applications.

## Introduction

Chronic kidney disease (CKD), characterized by high prevalence, low awareness, low treatment rates, poor control, adverse outcomes, and high medical costs, has become a significant public health issue severely impacting human health and quality of life (1). Due to the insidious onset of CKD and lack of patient awareness, many patients were late referral to until the disease has advanced to end-stage renal disease (ESRD). In recent years, the incidence of ESRD in China has been increasing annually (2). Peritoneal dialysis (PD), with its simplicity, safety, effectiveness, and suitability for home treatment, has gained widespread use globally, especially in developing countries, including China (3).

However, a considerable proportion of PD patients suffer from malnutrition, exacerbating various metabolic disorders and significantly increasing the risk of death and hospitalization (4). The prevalence of malnutrition among PD patients ranges from 11.7 to 47.8% (5, 6).

Nutritional therapy is essential for improving complications such as the micro-inflammatory state, anemia, and bone mineral metabolism abnormalities in dialysis patients. Thus, addressing the nutritional issues of PD patients and integrating nutritional therapy throughout the treatment process is crucial for enhancing overall diagnostic and treatment levels, delaying disease progression, improving patient outcomes, and reducing healthcare costs (7, 8).

The 2020 Kidney Disease Outcomes Quality Initiative (KDOQI) Clinical Practice Guidelines for Nutrition in Chronic Kidney Disease (Updated Version) recommend a daily dietary protein intake of 1.0–1.2 g/kg body weight for metabolically stable adult PD patients to maintain stable nutritional status (9, 10). The “Chinese Clinical Practice Guidelines for Nutritional Therapy of Chronic Kidney Disease 2021” recommends a protein intake of 1.0–1.2 g·kg^−1^·d^−1^ for patients without residual renal function and 0.8–1.0 g·kg^−1^·d^−1^ for those with residual renal function, with over 50% of the protein intake consisting of high biological value proteins (11). However, traditional dietary management focuses on controlling intake, which, although crucial for maintaining patient health, often lacks personalization and is difficult to implement, making it challenging to accommodate specific lifestyle habits and preferences, resulting in poor patient compliance.

In recent years, artificial intelligence (AI) technology has demonstrated tremendous potential in medical education, patient management, particularly in providing personalized medical care (12–15). The advent of AI-driven tools such as ChatGPT presents an innovative method for managing diets in patients with ESRD who are undergoing dialysis (16). Previous research showed that using the GPTs feature of ChatGPT to assist patients in dietary management effectively controlled the blood potassium levels of dialysis patients (17). To further expand the application of AI in the management of PD, we aim to develop a smart recipe generation tool that precisely controls protein intake while considering individual tastes and dietary preferences, offering customized dietary management plans. This tool, based on GPT technology, can learn from a vast array of CKD dietary guidelines to generate personalized recipes tailored to the needs of PD patients.

In this study, we used a self-controlled design to evaluate the impact of an AI-based recipe generation tool on the nutritional status of PD patients. This study not only aim to provide a new solution for the daily management of PD patients but also opens new pathways for using technology to improve overall health management in patients with chronic diseases, having significant clinical implications.

## Methods

### Development of the GPT-based recipe generation tool

This study utilized a customized version of the GPT-4 model (https://chat.openai.com/g/g-3ljI7scae-fu-tou-huan-zhe-yin-shi-zhi-nan),whichwas fine-tuned based on the Chinese Kidney Diet Guidelines (11), the 2020 KDOQI Nutrition Guidelines (9, 10), and the Mayo Clinic’s Kidney Diet Handbook. This ensured that the generated recipes met the specific nutritional needs of PD patients. During the inference process, we used these resources as a Retrieval-Augmented Generation (RAG) knowledge base. The model’s hyperparameters, such as temperature (set to 0.7) and top-p (set to 0.9), were adjusted, and the prompt incorporated patients’ dietary habits and individual characteristics as inputs.

The tool analyzes patients’ food preferences and nutritional requirements (especially regarding protein and phosphorus control), using GPT technology to generate personalized meal plans that meet individual needs. Additionally, the tool can adjust recommendations based on patient feedback to optimize nutritional intake balance.

### Patient recruitment and data collection

This study recruited 35 ESRD patients undergoing PD at our center between January and February 2024. Inclusion criteria encompassed patients aged ≥18 years who had been receiving PD treatment for at least 3 months. Exclusion criteria included patients with severe, life-threatening complications such as myocardial infarction, severe infections, or advanced malignancies, as well as those with eating disorders. The sample size was determined based on an assumed medium effect size (Cohen’s *d* = 0.5). We set the significance level *α* at 0.05 and the statistical power (1-*β*) at 0.8, resulting in a calculated minimum sample size of 32 participants. To ensure the representativeness of the study and the reliability of the results, we expanded the sample size to 35 participants.

### Intervention procedure

The study involved a two-phase dietary intervention. In the initial phase, patients received standard dietary education provided by professional renal dietitians. The educational content was based on the KDOQI Nutrition Guidelines and Chinese Clinical Practice Guidelines for Kidney Disease, covering topics such as protein intake, phosphate management, and fluid-electrolyte balance. Patients were also given detailed dietary materials to help them understand how to adjust their diet according to their individual dialysis needs. Following this, their serum prealbumin, serum albumin, and blood phosphorus levels were measured. In the second phase, doctors generated personalized weekly meal plans for the patients based on their weight, residual kidney function, and dietary preferences. All patients received training on how to provide their dietary preferences to the doctors and how to interpret the feedback. During weekly doctor visits, meal plans were adjusted according to patient feedback. All menus created by the doctors were reviewed by nutrition experts to ensure they met the patients’ clinical needs. After 4 weeks of using this tool, their serum prealbumin, serum albumin, and blood phosphorus levels were reassessed. During the follow-up period, no new medications affecting appetite were added or discontinued.

### Case presentation

**Figure 1:**
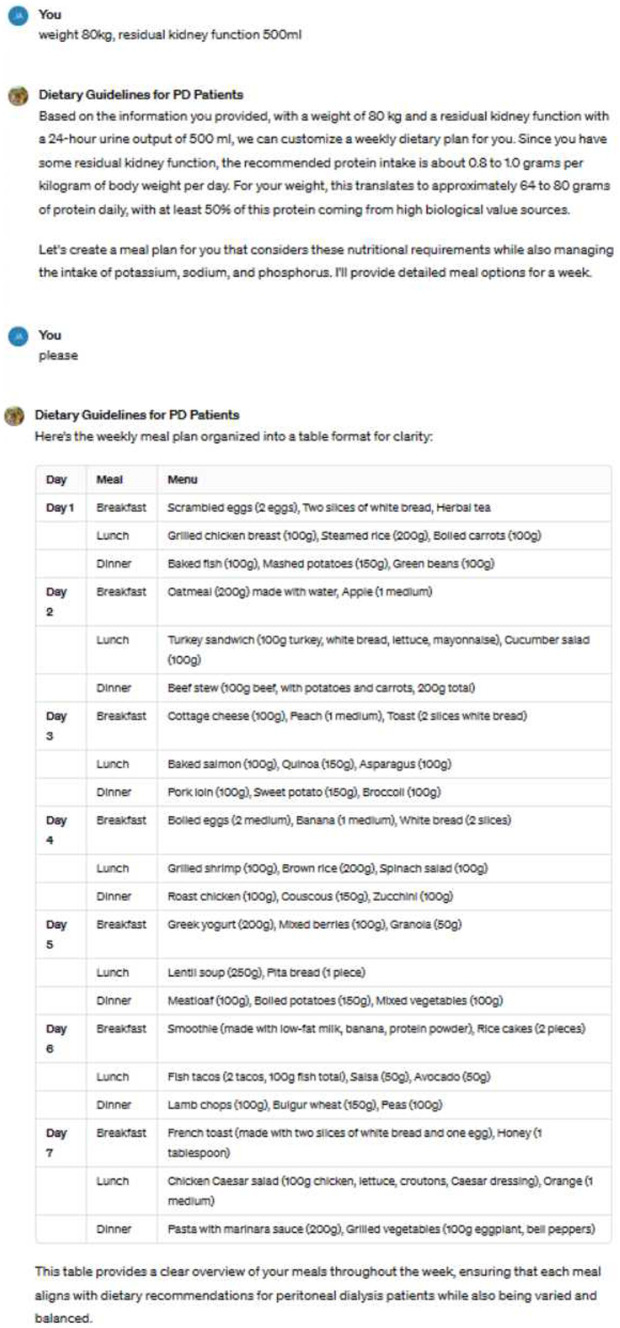
An example of the dialogue used in our study to illustrate the specific content.

The image illustrates an example of us utilizing ChatGPT to guide patients’ dietary choices (Figure 1). Based on the patient’s weight, residual kidney function, and dietary preferences, the tool generates recommended weekly meal plans.

### Statistical analysis

The statistical analysis was performed using SPSS 26.0 software. In the analysis, all continuous data are presented as mean ± standard deviation (for normally distributed data) or median and interquartile range (for non-normally distributed data). Categorical data are described using frequencies and percentages. To evaluate the impact of different dietary guidance strategies on patients’ laboratory indicators, we employed a mixed-effects linear regression model. This model was carefully selected to adequately account for both fixed effects (representing the dietary advice) and random effects (explaining inter-individual variability among patients). The “statsmodels” library in Python served as our primary tool for conducting the statistical analysis. For comparing categorical data between groups, we utilized the chi-square test. A significance level of *p* < 0.05 was established for all statistical tests to ensure the rigor and reliability of our research findings.

## Results

### Overall participant characteristics

This study included a total of 35 patients undergoing PD. All participants utilized the recipe generation tool during the study period and had their laboratory indicators assessed before and after the intervention (Table 1).

**Table 1:** Baseline characteristics.

Feature	*N* = 35
Age (years)	54.0 (39.5–67.5)
Dialysis age (months)	26.0 (15.0–40.0)
White blood cell count (10^9/L)	6.06 (5.00–7.23)
Hemoglobin (g/L)	113.60 ± 19.81
Platelet count (10^9/L)	199.86 ± 64.97
Prealbumin (mg/L)	289.04 ± 74.60
Albumin (g/L)	34.70 ± 5.94
Phosphorus (mmol/L)	1.45 ± 0.33
Calcium (mmol/L)	2.20 (2.04–2.32)
PTH (pg/mL)	333.16 ± 212.37
Low-density lipoprotein (mmol/L)	2.09 ± 0.63
High-density lipoprotein (mmol/L)	1.05 (0.92–1.35)
Total cholesterol (mmol/L)	4.11 (3.61–4.46)
ALT (U/L)	12.60 (9.40–18.45)
Alkaline phosphatase (U/L)	89.00 (74.50–127.00)
Ferritin (μg/L)	137.10 (58.40–191.15)
Transferrin saturation (%)	30.00 (22.16–41.51)
CRP (mg/L)	2.82 (1.15–9.54)
BNP (pg/mL)	104.10 (55.10–196.30)
Cardiothoracic ratio	0.57 ± 0.07
kt/v	1.96 (1.77–2.30)
Ccr	58.63 (49.00–88.42)

PTH, parathyroid hormone; ALT, alanine transaminase; CRP, C-reactive protein; BNP, brain natriuretic peptide; Ccr, creatinine clearance.

### Changes in serum prealbumin levels

After receiving conventional dietary advice adhering to standard guidelines, patients exhibited a mean serum prealbumin level of 289.04 ± 74.60 mg/L. Moreover, following dietary guidance based on GPT recommendations, patients exhibited a significant higher mean serum prealbumin level of 326.72 ± 78.89 mmol/L. In this study, by applying a mixed-effects linear regression model analysis, it was found that the dietary intervention method had a significant impact on patients’ prealbumin levels (*p* = 0.001), with an average increase of 37.69 ± 11.48 mg/L.

**Figure 2:**
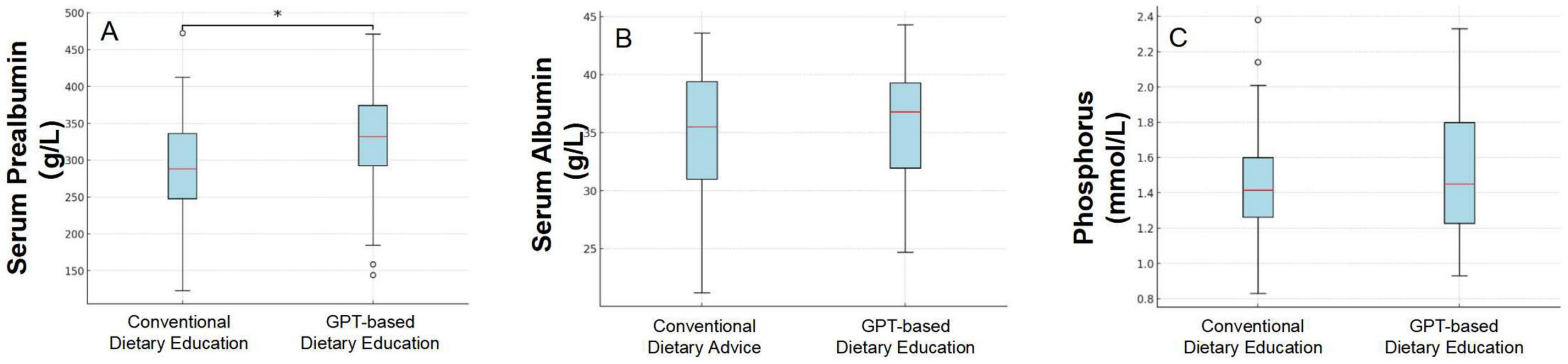
Comparison of three indicators (Serum Prealbumin levels, Serum Albumin levels, and Serum Phosphate) before and after dietary intervention based on conventional advice and GPT-guided recommendations.

Patients adhering to conventional dietary recommendations exhibited normal serum prealbumin levels—defined as serum albumin exceeding 300 mg/L—in 42.86% of instances. In contrast, following dietary guidance derived from GPT recommendations led to a significant increase in the proportion of patients with normal serum prealbumin levels, reaching 71.43% (*p* = 0.03) (Figure 2).

### Changes in serum albumin levels

After adhering to conventional dietary advice that aligns with standard guidelines, patients displayed an average serum albumin level of 34.70 ± 5.94 g/L. However, after implementing dietary guidance informed by GPT recommendations, the average serum albumin level in patients increased slightly to 35.66 ± 5.14 g/L. In this study, we employed a mixed-effects linear regression model to evaluate the impact of an intervention on patients’ albumin levels. The model results indicated that, after accounting for individual differences, the mean change in albumin concentration before and after the intervention had a coefficient of 0.97, with a standard error of 0.68. Although there was an increasing trend in albumin levels following the intervention, this change was not statistically significant (*p* = 0.153).

### Changes in serum phosphate levels

We compared the blood phosphorus levels of PD patients before and after a dietary intervention. The analysis showed that the average blood phosphorus level before the intervention was 1.45 ± 0.33 mmol/L; after the intervention, the average level was 1.52 ± 0.36 mmol/L. Using a mixed effects linear regression model, the results showed that the GPT-based dietary intervention led to an average increase in phosphate levels of 0.07 mmol/L, with a standard error of 0.06 mmol/L. No statistical difference was observed (*p* = 0.241).

## Discussion

In this study, it was found that the implementation of a GPT-based recipe generation tool notably enhanced serum prealbumin levels. It also moderately improved serum albumin levels while ensuring the stability of serum phosphate levels among PD patients. These results emphasize the potential and efficacy of technological interventions in optimizing nutritional management for patients with chronic conditions.

The improvement in serum prealbumin level and serum albumin level are important indicators of enhanced nutritional status (18, 19). In our study, patients showed a significant increase in serum prealbumin levels after using personalized meal plans. This outcome can be attributed to several factors: firstly, the AI-based recipe tool calculates the daily protein requirements precisely according to Chinese guidelines for the nutritional management of PD patients, ensuring patients receive adequate high-quality protein, which is crucial for PD patients; secondly, the personalized design of the meal plans considers patients’ dietary habits and food accessibility, enhancing patient adherence and making it easier for them to maintain healthy eating habits.

PD patients have strict protein management requirements, and improper management of protein intake can cause fluctuations in serum phosphate levels (20–22). Therefore, when developing dietary plans, it is important to choose foods with a low phosphate-to-protein ratio and low phosphate absorption rates, while limiting intake of foods high in phosphate additives (23). Previous research has also demonstrated the capability of AI tools in managing related indicators. In the recipe generation process, the GPT-4 tool paid particular attention to controlling phosphorus intake. By selecting foods with low phosphorus-to-protein ratios and low phosphorus absorption rates, such as fish and eggs, the tool avoided excessive phosphorus intake. The recipe generation tool successfully avoided electrolyte imbalances that could arise from excessive intake while ensuring adequate nutrient intake, which is especially critical for PD patients.

This study highlights the potential applications of AI technology in chronic disease management. Utilizing big data, pre-trained models, and machine learning algorithms, the recipe tool is able to provide precise nutritional recommendations, a feat often challenging to achieve with traditional nutritional guidance. At the end of the study, we collected patient feedback on their experience using the GPT-4 tool. Most patients reported that the tool generated meal plans that aligned with their tastes and cultural backgrounds, while also providing nutritional advice that was easy to follow. The personalized recommendations of the tool may also enhance patient satisfaction and adherence, aspects often lacking in traditional methods.

Despite the encouraging results, this study still has some limitations. A significant limitation of this study is the lack of a comprehensive assessment of patients’ actual nutrient intake and adherence to the GPT-generated meal plans. As a result, while we observed improvements in prealbumin levels, it cannot be conclusively attributed solely to the intervention, as actual nutrient intake was not systematically recorded. Another limitation of this study is the absence of a control group. While the self-controlled design allowed us to compare pre- and post-intervention data within the same patients, it limits our ability to draw definitive conclusions about the intervention’s effectiveness. Without a parallel control group, it is difficult to rule out the influence of external factors on the observed outcomes. Future studies should include a randomized controlled trial design to more accurately assess the efficacy of the intervention. Additionally, as a pilot study, the relatively small sample size and short study duration may limit the generalizability and sustainability of the observed effects. The intervention period of only 1 month may also be insufficient to capture long-term nutritional improvements. Due to the restrictions on using ChatGPT in China, which may cause inconvenience in practical applications, we have further developed the software by calling APIs to ensure that more patients can use it conveniently.

Future research could consider applying this smart recipe generation tool to other types of chronic disease patients, such as those with diabetes or cardiovascular diseases, to assess its applicability and effectiveness in broader chronic disease management. Additionally, exploring the integration of this technology with other health management tools, such as AI-based exercise plan generators and wearable devices for symptom monitoring, could provide a more comprehensive health management solution.

## Conclusion

Overall, the GPTs system offers a significant advancement in the dietary management of PD patients by enhancing their nutritional status. Its precise menu generation, tailored to both nutritional needs and patient preferences, along with demonstrated clinical improvements, underscores its value as a supplementary resource to conventional dietary counseling. With additional enhancements and full integration, AI-powered tools like the GPTs system could transform dietary management in PD and possibly other conditions sensitive to diet.

## Practical application

By leveraging pre-learned relevant knowledge and employing advanced content generation capabilities of large language models, our designed ChatGPT tool can generate menus tailored to the nutritional status of PD patients based on their dietary preferences. This innovative feature supports patients by calculating the required daily protein intake based on their provided weight and residual kidney function, and generating corresponding menus. This is crucial for patients managing their diet during PD. The tool has significant potential in the dietary management of ESRD patients, effectively improving their nutritional status.

## Data Availability

The original contributions presented in the study are included in the article/supplementary material, further inquiries can be directed to the corresponding authors.
